# A Self-Healing Gel with an Organic–Inorganic Network Structure for Mitigating Circulation Loss

**DOI:** 10.3390/gels10020093

**Published:** 2024-01-25

**Authors:** Cheng Wang, Jinsheng Sun, Yifu Long, Hongjun Huang, Juye Song, Ren Wang, Yuanzhi Qu, Zexing Yang

**Affiliations:** 1 College of Chemistry and Chemical Engineering, Southwest Petroleum University, Chengdu 610500, China; wangchengscc@163.com; 2 CNPC Engineering Technology R&D Co., Ltd., Beijing 102206, China; 3 CNPC Great Wall Drilling Engineering Co., Ltd., Beijing 102206, China

**Keywords:** drilling fluid, layered double hydroxide, self-healing gel, nanocomposite gels, lost circulation control

## Abstract

Lost circulation control remains a challenge in drilling operations. Self-healing gels, capable of self-healing in fractures and forming entire gel block, exhibit excellent resilience and erosion resistance, thus finding extensive studies in lost circulation control. In this study, layered double hydroxide, Acrylic acid, 2-Acrylamido-2-methylpropane sulfonic acid, and CaCl_2_ were employed to synthesize organic-inorganic nanocomposite gel with self-healing properties. The chemical properties of nanocomposite gels were characterized using X-ray diffraction, Fourier transform infrared spectroscopy, scanning electron microscope, X-ray photoelectron spectroscopy and thermogravimetric analysis. layered double hydroxide could be dispersed and exfoliated in the mixed solution of Acrylic acid and 2-Acrylamido-2-methylpropane sulfonic acid, and the swelling behavior, self-healing time, rheological properties, and mechanical performance of the nanocomposite gels were influenced by the addition of layered double hydroxide and Ca^2+^. Optimized nanocomposite gel AC_6_L_3_, at 90 °C, exhibits only a self-healing time of 3.5 h in bentonite mud, with a storage modulus of 4176 Pa, tensile strength of 6.02 kPa, and adhesive strength of 1.94 kPa. In comparison to conventional gel, the nanocomposite gel with self-healing capabilities demonstrated superior pressure-bearing capacity. Based on these characteristics, the nanocomposite gel proposed in this work hold promise as a candidate lost circulation material.

## 1. Introduction

Lost circulation refers to the phenomenon in drilling operations where drilling fluid within the well-bore leaks into the formation under the influence of pressure differentials [1,2,3,4]. Lost circulation not only causes delays in drilling operations and extends drilling cycles but also leads to issues such as well collapse, differential sticking, and even serious drilling accidents such as blowouts [5,6,7]. Traditional bridging materials widely used to address lost circulation problems have certain limitations, including poor compatibility with fractures and difficulty in forming high-strength plugging layers. Therefore, there is a need to develop new lost circulation material (LCM) with strong adaptability and excellent resistance to scouring [8,9].

Self-healing gel is a type of polymer material that can self-heal after being damaged, partially or completely restoring its original properties [10]. Self-healing gels can continuously expand, accumulate, and ultimately self-heal to form an entire gel block, achieving the goal of plugging fractures [11]. Compared to traditional bridging materials, self-healing gels have advantages such as good matching and stability in forming plugging layers [9]. Research on self-healing gels has been conducted in enhancing oil recovery and lost circulation control [12,13,14,15,16]. However, challenges such as the strength of self-healing gels in fractures and self- healing time for self-healing still need to be addressed [13,14,17,18]. Shortening the self-healing time of the self-healing gel can reduce non-productive time (NPT) and reduce the cost of construction. Layered double hydroxide (LDH) is an inorganic compound composed of negatively charged anions and positively charged metal hydroxides [19,20,21]. Due to their tunable chemical composition and excellent physical properties, LDH have been used as cross-linking agents in the preparation of nanocomposite (NC) gels [22,23,24]. To obtain uniform hydrogels, LDH used as cross-linking agents need to be pre-exfoliated or modified to form dispersed nanosheets [25,26]. Chen et al. prepared LDH/polyacrylamide NC hydrogels by stripping LDH monolayer nanosheets and using them as cross-linking agents [27]. Zhang et al. directly synthesized monolayer LDH nanosheets and prepared NC hydrogels in situ together with *N*-isopropylacrylamide monomer [28]. Currently, research on LDH/polymer NC hydrogels had mostly focused on introducing LDH nanosheets into hydrogels to enhance mechanical performance and stability [25,28]. However, there are few studies on utilizing the reversible dynamic cross-linking between LDH and polymers to form self-healing gels. The positively charged layer of LDH and the abundant hydroxyl groups on the surface can form reversible physical cross-linking such as electrostatic interaction and hydrogen bonding with polymers containing anionic groups. Therefore, the introduction of nanomaterial LDH could not only improve the mechanical properties of the gel but also form a gel with rapid self-healing through reversible dynamic bonds.

To avoid the disadvantages of low strength of traditional preformed particle gel and uncontrollable gelation time of in situ formed gels, in this study, LDH was dispersed in the mixed solution of Acrylic acid (AA) and 2-Acrylamido-2-methylpropane sulfonic acid (AMPS), utilizing the dynamic cross-linking properties between LDH and copolymer of AA-AMPS (P(AA-AMPS)) to synthesize NC gels with self-healing properties. The chelation interaction between Ca^2+^ and the anion functional group was introduced to further enhance the mechanical properties. The LDH/P(AA-AMPS) NC gels exhibited excellent mechanical and self-healing properties, demonstrating superior pressure resistance compared to conventional gels. Different from the traditional LDH NC gel, the introduction of LDH in LDH/P(AA-AMPS) NC gels not only improves the performance of the gel but also endows the gel with self-healing properties as a dynamic cross-linking agent. Thus, LDH/P(AA-AMPS) NC gels provide a novel LCM for lost circulation control.

## 2. Results and Discussion

### 2.1. Synthesis of LDH/P(AA-AMPS) NC Gels

**Figure 1:**
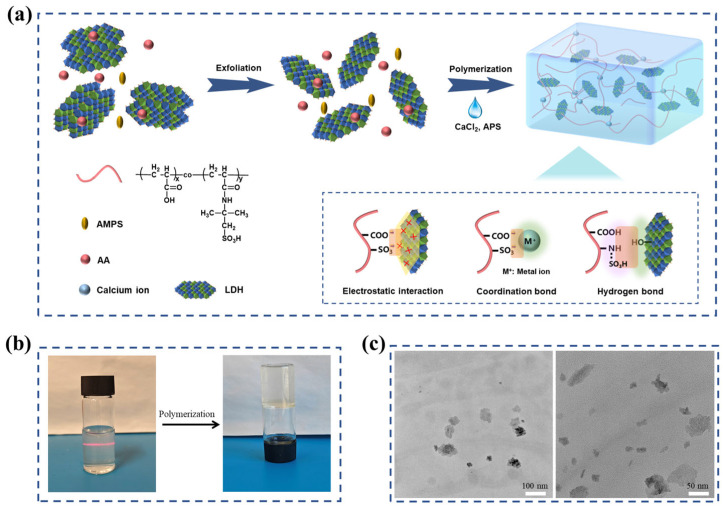
(**a**) Schematic illustration of LDH/P(AA-AMPS) NC gels. (**b**) Photographs of AC_6_L_3_ before and after polymerization. (**c**) TEM images of LDH dispersed in AA-AMPS solution.

The synthesis of LDH/P(AA-AMPS) NC gels involved two main steps: the dispersion of LDH and the monomer polymerization, as depicted in Figure 1a. LDH appeared transparent in the AA-AMPS solution with a distinct Tyndall effect (Figure 1b), indicating that the solution hadcolloidal properties [26,29]. As shown in Figure 1c, the transmission electron microscope (TEM) test of LDH in AA-AMPS solution showed a low contrast, indicating that LDH was partially exfoliated in AA-AMPS solution [28]. This was attributed to the ion exchange between COO^−^, SO_3_^−^, and CO_3_^2−^ of LDH. Subsequently, in situ formation of P(AA-AMPS) was initiated by APS adsorbed on LDH [26]. The LDH/P(AA-AMPS) NC gels were cross-linked through electrostatic interactions, coordination interactions, and hydrogen bonding. Additionally, chelation structures formed by the metal ion with carboxyl groups on the P(AA-AMPS) molecular chains further enhanced the intermolecular cross-linking of the gel.

### 2.2. Characterization of LDH/P(AA-AMPS) NC Gels

**Figure 2:**
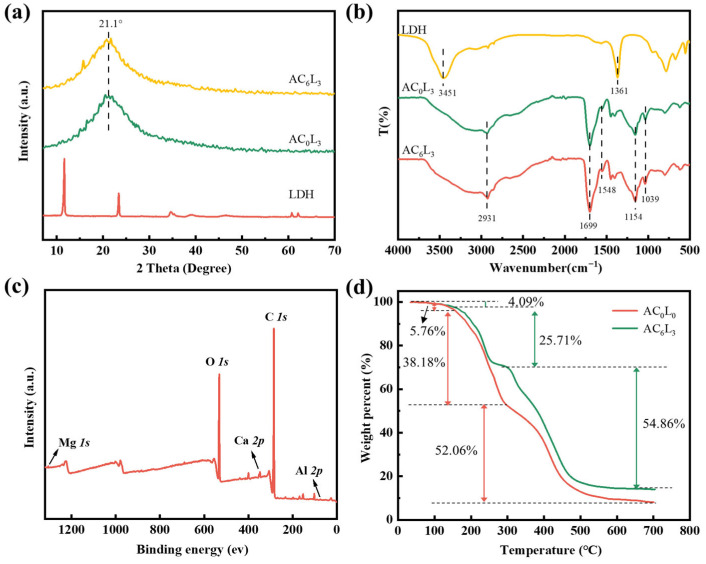
(**a**) XRD patterns of the different samples. (**b**) FT-IR spectra of the different samples. (**c**) XPS survey spectra of AC_6_L_3_. (**d**) TGA curves of AC_0_L_3_ and AC_6_L_3_.

X-ray diffraction (XRD) is a typical method for characterizing crystal structures. The XRD patterns of samples with LDH are shown in Figure 2a. The peaks around 21.1° of AC_6_L_3_ and AC_0_L_3_ were attributed to the amorphous P(AA-AMPS) [30]. Furthermore, AC_6_L_3_ and AC_0_L_3_ exhibited no characteristic diffraction peaks of LDH, indicating the absence of a large number LDH with ordered structure in the gel [28]. Fourier transform infrared spectroscopy (FT-IR) characterization of functional groups in LDH/P(AA-AMPS) NC gels is presented in Figure 2b. The LDH showed characteristic bands at 3451 and 1361 cm^−1^, which corresponded to the bending vibration of –OH and the CO_3_^2−^ in the LDH interlayer [31]. For AC_0_L_3_ and AC_6_L_3_, the absorption peak at 2931 cm^−1^ corresponded to the stretching vibration peak of C–H. The absorption peaks at 1699 cm^−1^ and 1548 cm^−1^ were attributed to the stretching vibration of C=O and the bending vibration peak of N–H, respectively. The absorption peaks at 1154 cm^−1^ and 1039 cm^−1^ represented the infrared characteristic absorption peak of –SO_3_H and the vibration absorption peak of C–H, respectively [32]. Compared with LDH, the –OH and CO_3_^2−^ peaks of AC_0_L_3_ and AC_6_L_3_ at 3451and 1361 cm^−1^ disappeared, indicating that LDH dispersed in the AA-AMPS solution participated in the cross-linking of the gel network [30,31,33].

X-ray photoelectron spectroscopy (XPS) characterization of AC_6_L_3_ samples was conducted to determine the elemental composition and chemical states. The full spectrum revealed the presence of C, O, Ca Mg, and Al elements in AC_6_L_3_, as seen in Figure 2c. Figure S1 shows the high-resolution spectra of C 1s, O 1s, and Ca 2p. The high-resolution C 1s spectrum at 284.8 eV, 286.3 eV and 288.7 eV corresponded to C–C, C–O, and O=C–O. Peaks at 530.7 eV, 532.1 eV, 533.5 eV, and 534.3 eV in the O 1s spectrum were attributed to metal-O, C–O, C=O, and O–H [34]. For Ca 2p, the peaks at 346.9 eV and 350.5 eV corresponded to the characteristic peaks of COO–Ca [30]. These results indicate the successful incorporation of inorganic nanomaterial LDH and Ca^2+^ into the gel network.

To investigate the thermal stability of LDH/P(AA-AMPS) NC gels, thermogravimetric analysis (TGA) testing was conducted on AC_0_L_0_ and AC_6_L_3_ under a nitrogen atmosphere (Figure 2d). The TG curve showed that the weight loss of AC_0_L_0_ and AC_6_L_3_ was divided into three stages of mass loss. For AC_6_L_3_, the weight loss from room temperature to168 °C was 4.09%, attributed to the removal of absorbed and bonded water. From 168 °C to 305 °C, the 25.71% weight loss involved dehydroxylation of LDH, decomposition of carboxyl groups on the molecular chain. Beyond 305 °C, the main chain of sulfonic groups, the destruction of cross-linked network structure, and the layered structure of LDH were disrupted [35,36]. The weight losses of AC_0_L_0_ in the three weight loss stages were 5.76%, 38.18%, and 52.06%, respectively. In addition, the residual amount of AC_6_L_3_ was higher than that of AC_0_L_0_. This indicates that AC_6_L_3_ exhibited superior stability with the introduction of LDH and Ca^2+^.

**Figure 3:**
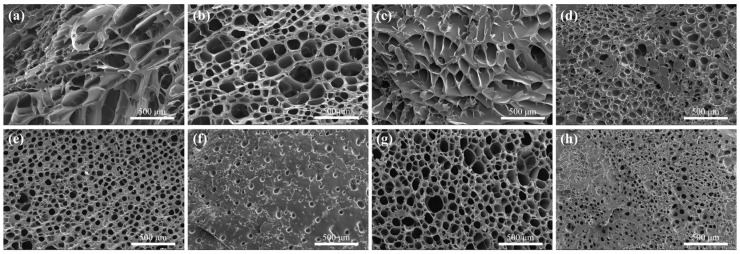
SEM images of different samples: (**a**) AC_0_L_0_, (**b**) AC_0_L_3_, (**c**) AC_6_L_0_, (**d**) AC_6_L_3_, (**e**) AC_6_L_1.5_, (**f**) AC_6_L_4.5_, (**g**) AC_3_L_3_, (**h**) AC_9_L_3_.

The microstructures of LDH/P(AA-AMPS) NC gels were investigated and scanning electron microscope (SEM) images of the samples after freeze–drying are presented in Figure 3. AC_0_L_3_ sample exhibited a porous and interconnected three-dimensional network structure, while the AC_3_L_3_ sample had denser pore sizes. With increasing Ca^2+^ content, pore sizes gradually decreased, indicating increased cross-linking. This was attributed to the chelation structure formed by COOH on P(AA-AMPS) polymer chains coordinating with Ca^2+^, enhancing the cross-linking of the samples [37]. Compared to AC_6_L_0_, AC_6_L_1.5_ showed thicker pore walls and fewer pores. Moreover, with an increase in LDH content, pore sizes decreased, and pore walls thickened. This was due to the electrostatic, coordination, and hydrogen bonding interactions between LDH and P(AA-AMPS), enhancing the cross-linking of the samples [28,29].

### 2.3. Self-Healing Properties of LDH/P(AA-AMPS) NC Gels

**Figure 4:**
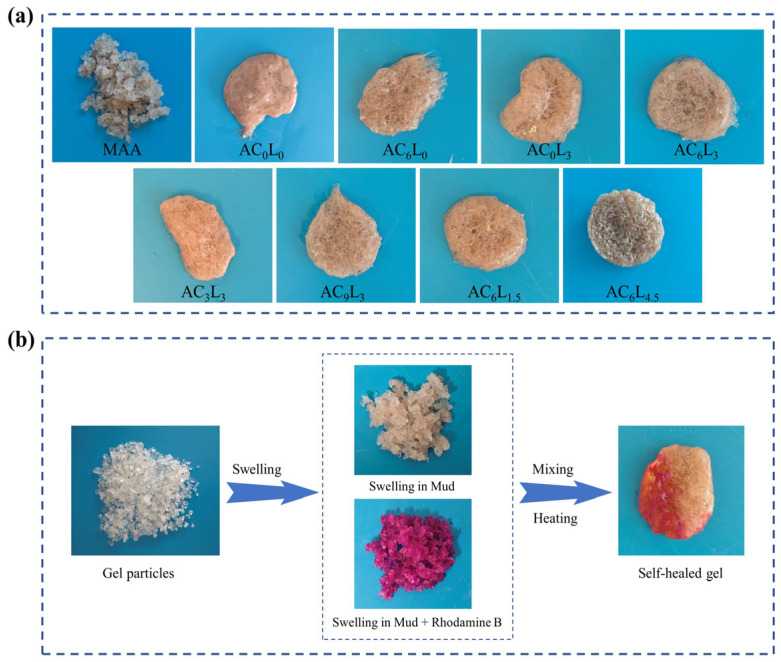
(**a**) Photographs of different self-healed gels. (**b**) Photographs of AC_6_L_3_ before and after self-healing.

**Figure 5:**
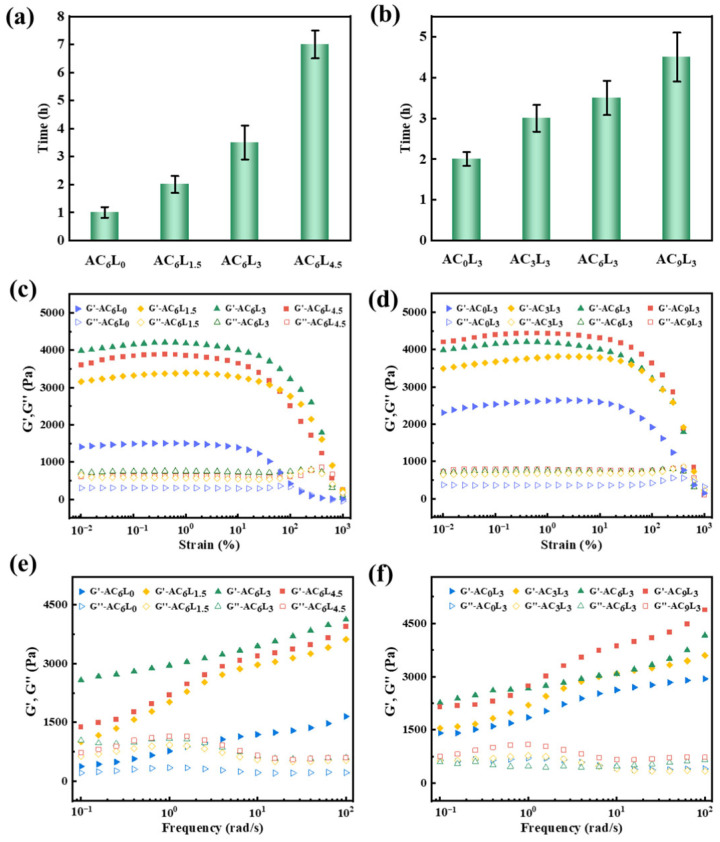
(**a**,**b**) Self-healing time of the different samples. (**c**,**d**) Dynamic strain sweeps of different self-healed gels. (**e**,**f**) Dynamic frequency sweeps of different self-healed gels.

The self-healing performance of LDH/P(AA-AMPS) NC gels was the key factor affecting the pressure bearing capacity. Thus, the self-healing performance of LDH/P(AA-AMPS) NC gels was tested by adding a 10% concentration of LDH/P(AA-AMPS) NC gels particles into bentonite mud. As shown in Figure 4a, at 90 °C, MAA remained as independent gels after expansion, unable to self-heal. AC_0_L_0_ has obvious fluidity and cannot fix the shape, while AC_6_L_0_, AC_0_L_3_ and AC_6_L_3_ were able to self-heal into an entire gel after water absorption and expansion in bentonite mud. Taking AC_6_L_3_ as an example, the expanded AC_6_L_3_ gel in bentonite mud and bentonite slurry + rhodamine obviously became an entire gel composed of different colors after self-healing, which further proved the self-healing properties (Figure 4b). However, as shown in Figure 5a, the self-healing time of AC_6_L_1.5_ at 90 °C was 2 h, longer than the 1 h for AC_6_L_0_. As the amount of LDH added was 3 g, the self-healing time was extended to 3.5 h. Moreover, as the LDH content increased, the self-healing time of AC_6_L_9_ was further extended to 7 h. At the same time, the self-healing time of AC_0_L_3_ and AC_3_L_3_ was 2 h and 3 h, respectively. Further increasing the CaCl_2_ addition to 9 g extended the self-healing time to 4.5 h (Figure 5b). The introduction of LDH and Ca^2+^ increased the cross-linking density of the gel, restricting the movement of molecular chains [38]. Furthermore, excessive LDH led to shorter polymer chain lengths in the cross-linked polymer, resulting in reduced healing efficiency [39,40].

Rheological and mechanical strengths are crucial indicators for evaluating the stability of gels in fractures. Therefore, the rheological and mechanical properties of the gel after self-healing at 90 °C were tested. Figure 5c,d shows the strain sweep curves of the self-healed gel in the range of 0% to 1000%. In the early stage of strain increase, the storage modulus (G′) was higher than the loss modulus (G″), indicating predominantly elastic solid characteristics and a stable gel network [41,42]. However, as the strain continued to increase, the G′ gradually decreased, indicating the gradual breakdown of the gel structure [43]. In the linear viscoelastic region, the G′ value for AC_6_L_0_ was 1488 Pa, while the G′ values for AC_6_L_1.5_, AC_6_L_3_, and AC_6_L_4.5_ were 3305 Pa, 4176 Pa, and 3848 Pa, respectively (Figure S2). This suggests that with increasing LDH, the G′ value of self-healed gels initially increased and then decreased. This is attributed to the formation of an organic–inorganic NC gel and increased cross-linking degree through introducing LDH, enhancing the rheological strength, while excessive cross-linking negatively affects the rheological performance [27,28]. Additionally, increasing Ca^2+^ also improved the rheological performance of the self-healed gel; as the Ca^2+^ content increased from 3 g to 9 g, the G′ value of the self-healed gel increased from 3706 Pa to 4416 Pa (Figure S3). This is attributed to the chelation structure formed by Ca^2+^ with anionic groups on the P(AA-AMPS) chain [44]. Figure 5e,f presents the frequency sweep test results for the self-healed gels in the range of 0.1 to 100 rad/s. With increasing frequency, the G′ value of the self-healed gels showed a noticeable upward trend. This indicates that the difference between moduli changes with frequency, further demonstrating the dynamic cross-linking structure of LDH/P(AA-AMPS) NC gels [45,46].

**Figure 6:**
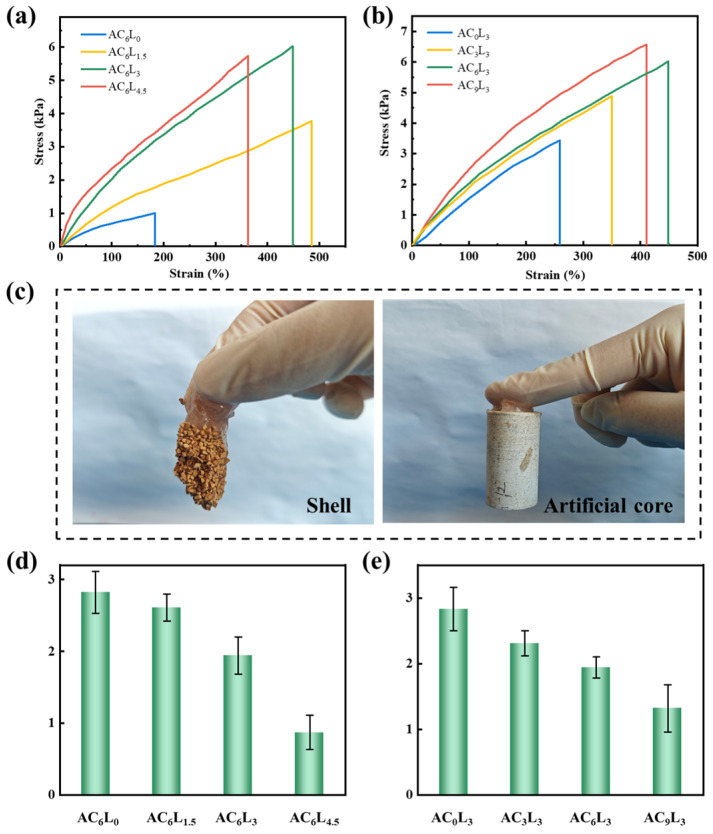
(**a**,**b**) Tensile stress–strain curves of different self-healed gels. (**c**) Adhesion of AC_6_L_3_ self-healed gel to shell and artificial core. (**d**,**e**) Adhesion strength of different self-healed gels to artificial core.

The tensile performance of the self-healed gels is shown in Figure 6a,b. As the LDH content increased from 0 to 3 g, the fracture strain of the self-healed gel gradually increased from 183% to 448%, and the fracture stress increased from 1.03 kPa to 6.02 kPa. However, further increasing the LDH amount to 4.5 g resulted in a decrease in fracture strain to 363%, with a decrease in fracture stress to 5.73 kPa. The reason for this change was that excessive LDH lead to over-cross-linking, thereby affecting mechanical properties [47,48]. Meanwhile, increasing Ca^2+^ from 0 to 9 also increased the fracture strain of the self-healed gel from 3.41 kPa to 6.56 kPa. This result was consistent with rheological testing and SEM characterization, further indicating that the introduction of LDH and Ca^2+^ increases the cross-linking density of LDH/P(AA-AMPS) NC gels, enhancing rheological and mechanical properties. As shown in Figure 6c, AC_6_L_3_ self-healed gel had adhesion properties to the shell and artificial core, which may be attributed to the hydrogen bonding between COOH and the surface of the material [49]. From the adhesion strength of different self-healed gels to artificial core, shown in Figure 6d,e, it can be found that the adhesion strength of AC_6_L_3_ self-healed gel was 1.94 kPa, and the adhesion strength of self-healed gel decreased with the increase in LDH and Ca^2+^. This was attributed to the introduction of LDH and Ca^2+^, resulting in a decrease in free carboxyl groups, thereby reducing the adhesion strength [50]. It also reflected the interaction between LDH, Ca^2+^ and P(AA-AMPS). Through a comprehensive comparison of self-healing time, rheological, mechanical and adhesion strength of different samples, AC_6_L_3_ was identified as the optimal sample and further evaluated.

**Figure 7:**
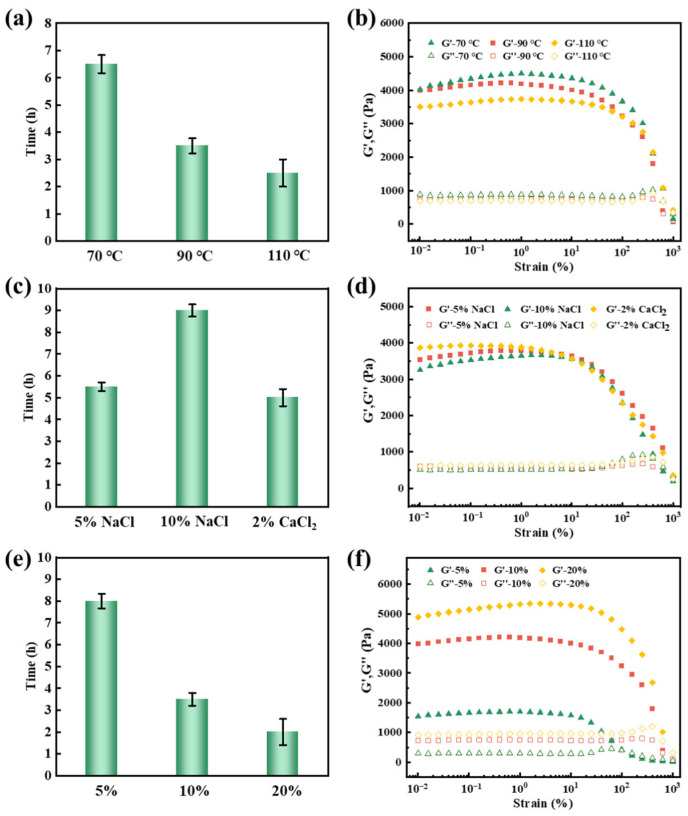
(**a**) Self-healing time of AC_6_L_3_ at different temperatures. (**b**) Dynamic strain sweeps of AC_6_L_3_ self-healed gels at different temperatures. (**c**) Self-healing time of AC_6_L_3_ at different brines. (**d**) Dynamic strain sweeps of AC_6_L_3_ self-healed gels at brines. (**e**) Self-healing time of different AC_6_L_3_ particle concentrations. (**f**) Dynamic strain sweeps of self-healed gels with different AC_6_L_3_ particle concentrations.

Temperature has a crucial effect on the properties of self-healing gels. The self-healing performance of AC_6_L_3_ was investigated at 70 °C, 90 °C, and 110 °C. As shown in Figure 7a, the self-healing time of AC_6_L_3_ at 70 °C was 6.5 h, and as the reaction temperature increased to 90 °C and 110 °C, the self-healing time of AC_6_L_3_ shortened to 3.5 h and 2 h, respectively. This was because, as the temperature rose, the migration rate of P(AA-AMPS) molecular chains, Ca^2+^, LDH between contacting gels increased, making P(AA-AMPS) molecular chains more prone to recombine with Ca^2+^ and LDH [51]. The G′ value of AC_6_L_3_ self-healed gel at 70 °C and 90 °C was 4251 Pa and 4176 Pa, respectively (Figure S4). However, with the reaction temperature increasing to 110 °C, the G′ value decreased to 3807 Pa (Figure 7b). This was attributed to the partial breakdown of the AC_6_L_3_ molecular chains’ structure at high temperatures, leading to a decrease in rheological performance. As drilling often encounters saline formations, it was essential to evaluate the self-healing performance of LDH/P(AA-AMPS) NC gels in brines. As shown in Figure 7c,d, the self-healing time of AC_6_L_3_ in 5% NaCl, 10% NaCl, and 2% CaCl_2_ was 5.5 h, 9 h, and 5 h, respectively, and the G′ values of self-healed gels were 3719 Pa, 3502 Pa, and 3917 Pa, respectively (Figure S5). Compared with the self-healing performance in salt-free bentonite mud, the self-healing time was prolonged, and the G′ value of self-healed gels was slightly decreased. This was attributed to the fact that the gel’s expansion rate in brines slowed down, and brines affected the coordination between the P(AA-AMPS) and LDH, Ca^2+^. The gel network was disrupted in brines, accelerating dissociation [52,53]. The self-healing performance of AC_6_L_3_ at different concentrations in bentonite mud was investigated and the result is shown in Figure 7e,f. As the AC_6_L_3_ gel particle concentration increased from 5% to 20%, the self-healing time decreased from 7.5 h to 2 h, and the G′ value of the self-healed gel increased from 1671 Pa to 5109 Pa (Figure S6).

### 2.4. Swelling Behavior of LDH/P(AA-AMPS) NC Gels

**Figure 8:**
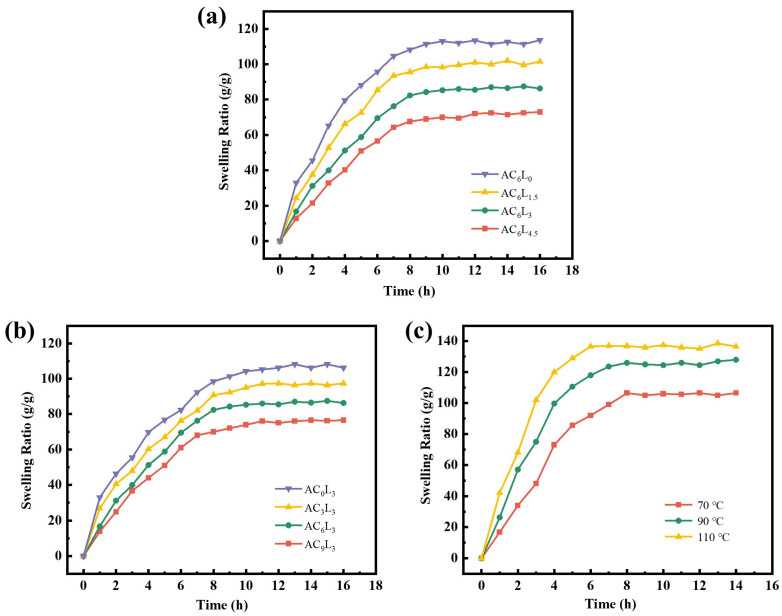
(**a**,**b**) Swelling behavior of different LDH/P(AA-AMPS) NC gel particles. (**c**) Swelling behavior of AC_6_L_3_ gel particle at different temperatures.

The swelling behavior of different LDH/P(AA-AMPS) NC gels particles in bentonite mud was tested. As shown in Figure 8a,b, the initial swelling degree of the gel rapidly increased, then gradually decreased, eventually reaching equilibrium. This was primarily due to the reorganization of dynamic bonds within the gel during the swelling process, leading to an increased cross-linking density and a more stable network structure [38]. Moreover, the equilibrium swelling ratio of AC_6_L_0_, AC_6_L_1.5_, AC_6_L_3_, and AC_6_L_4.5_ were 113.6 g/g, 101.5 g/g, 86.3 g/g, and 73.1 g/g, respectively. The equilibrium swelling ratio of AC_0_L_3_, AC_3_L_3_, AC_6_L_3_, and AC_9_L_3_ was 106.2 g/g, 97.4 g/g, 86.3 g/g, and 76.5 g/g, respectively. The equilibrium swelling ratio of LDH/P(AA-AMPS) NC gels decreased with increasing LDH and Ca^2+^. This phenomenon was attributed to the interactions between P(AA-AMPS) and LDH, Ca^2+^, resulting in a denser internal network structure of the gel that impedes water infiltration [22,27,54]. The swelling behavior of AC_6_L_3_ was investigated at different temperatures, and the results are presented in Figure 8c. The equilibrium swelling ratios of AC_6_L_3_ gel particles at 70 °C, 90 °C, and 110 °C were 106.5 g/g, 128.3 g/g, and 136.5 g/g, respectively. As the temperature increased, the equilibrium swelling ratio of AC_6_L_3_ gel particles gradually increased, accompanied by a reduction in the time to reach equilibrium swelling.

### 2.5. Plugging Performance of LDH/P(AA-AMPS) NC Gels

**Figure 9:**
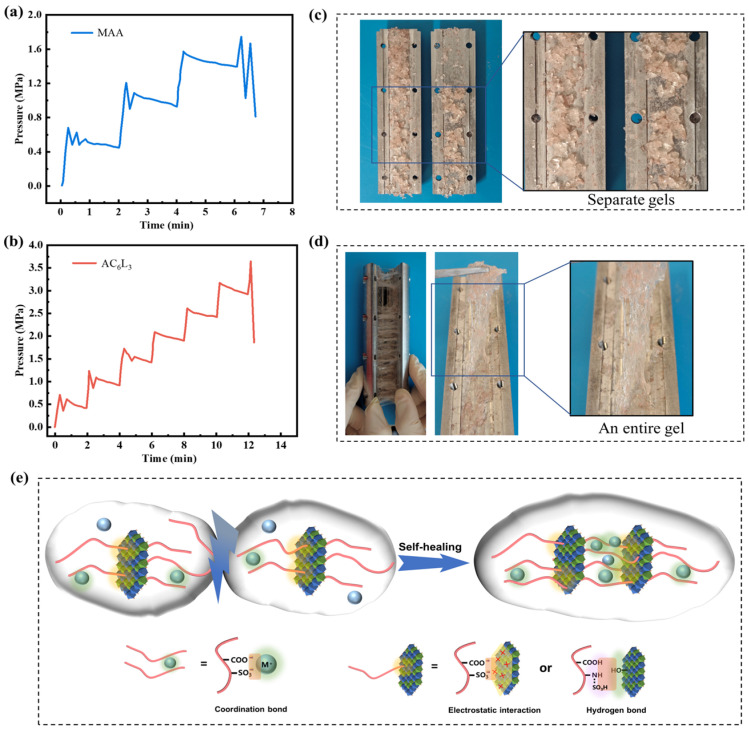
Plugging performance of LCM: (**a**) MAA, (**b**) AC_6_L_3_. The pictures of LCM in metal slit plate: (**c**) MAA was still separate gels, (**d**) AC_6_L_3_ was an entire gel. (**e**) Self-healing mechanism of LDH/P(AA-AMPS) NC gels.

The plugging performance is a critical parameter for evaluating LCM. Therefore, the metal slit plate was employed to assess the pressure-bearing capabilities of the LCM. Figure 9a,b showed the plugging properties of self-healing gel AC_6_L_3_ and conventional gel MAA. At 90 °C, the pressure-bearing capacity of AC_6_L_3_ reached 3 MPa, surpassing conventional gel MAA with a capacity of only 1.5 MPa. As illustrated in Figure 9c,d, AC_6_L_3_ exhibited the ability to adhere and self-heal, and form an entire gel block in the metal slit plate, while MAA was still an independent gel. Consequently, the self-healed entire gel block can more effectively fill and plug fractures, thereby enhancing the pressure-bearing performance of the LCM [11]. The plugging mechanism of the self-healing gel AC_6_L_3_ in formation fractures was deduced as follows: Initially, the deformable AC_6_L_3_, subjected to pressure differentials, enters the fractures and gradually expands and accumulates. Stimulated by the temperature of leakage layer, molecular chain movements intensify among the inter-contacted yet independent gel segments. P(AA-AMPS), LDH, and Ca^2+^ migrated between gel interfaces and progressively reassembled to form dynamic bonds under the cross-linking of electrostatic interactions, coordination interactions, and hydrogen bonding (Figure 9e), thereby self-healing into an entire gel block and achieving lost circulation control.

## 3. Conclusions

In summary, this study successfully synthesized self-healing LDH/P(AA-AMPS) NC gels by introducing LDH and Ca^2+^ into P(AA-AMPS). LDH and Ca^2+^ enhanced the thermal stability, rheological properties, and mechanical properties of self-healed gels. In addition, LDH and Ca^2+^ also prolonged the self-healing time of LDH/P(AA-AMPS) NC gels, slowed down the swelling rate, and reduced the adhesion strength. A comprehensive comparison of the performance of LDH/P(AA-AMPS) NC gels determined that the optimal sample had the self-healing time of 3.5 h, the G′ value of 4176 Pa, the swelling rate of 86.3 g/g, and the tensile strength of 6.02 kPa. As the temperature increased, the self-healing time of LDH/P(AA-AMPS) NC gels shortened, and the rheological strength of self-healed gels was slightly reduced. In brines, the self-healing time of LDH/P(AA-AMPS) NC gels was prolonged, with a slight decrease in the rheological strength after self-healing. As the concentration of gel particles increased, the self-healing time of self-healed gel shortened, and the rheological strength strengthened. The pressure-bearing capacity of LDH/P(AA-AMPS) NC gel was 3.5 MPa, surpassing the conventional gel’s pressure-bearing capacity of 1.5 MPa. In conclusion, the synthesized LDH/P(AA-AMPS) NC gels in this study provided a novel LCM with self-healing properties, offering a reference for Lost circulation control.

## 4. Experimental

### 4.1. Materials

Acrylic acid (AA, 98%), 2-Acrylamido-2-methylpropane sulfonic acid (AMPS, 99%), layered double hydroxide (LDH, 99%), *N*,*N*-methylenebisacrylamide bis-acrylamide (MBA, 99%), anhydrous calcium chloride (CaCl_2_, 96%), ammonium persulfate (APS, 99%), and sodium chloride (NaCl, 99.5%) were obtained from Aladdin Reagent Co., Ltd., Shanghai, China.

### 4.2. Preparation of LDH/P(AA-AMPS) NC Gels

The LDH/P(AA-AMPS) NC gel was synthesized through the free radical polymerization of monomers AA and AMPS. Specifically, 30 g of AA and 3 g of AMPS were dissolved in 100 mL of deionized water. After thorough stirring, 3 g of LDH was added, and the mixture was stirred for 5 h to achieve a homogeneous solution. Subsequently, 0.35 g of APS and 6 g of CaCl_2_ were added to the mixture, followed by an additional 3 h of stirring. The resulting homogeneous mixture was then placed in a 60 °C oven for 12 h to form LDH/P(AA-AMPS) NC gels. After the completion of the reaction, the obtained gel was cut into small pieces and dried at 60 °C for 48 h. Finally, the dried particles were crushed into particles with a particle size of 1~3 mm. For comparison, a series of gels were synthesized by varying the amounts of LDH and CaCl_2_ (Table 1). Additionally, a conventional gel (MAA) was synthesized using AA, AMPS, and the chemical cross-linking agent 0.5% MBA (wt % to the total mass of monomer).

### 4.3. Characterization

The functional groups in the LDH/P(AA-AMPS) NC gels were determined by FT-IR (WQF520, Beijing, China) with the assistance of KBr in a wavenumber range of 4000 to 500 cm^−1^. Morphology of LDH/P(AA-AMPS) NC gels were characterized using SEM (ZEISS EVO18, Oberkochen, Germany), and all the samples were freeze-dried before the measurement. The thermal stability of the samples was measured through TGA (NetzschSTA449F3, Selb, Germany) in a nitrogen atmosphere with a heating rate of 10 °C/min that changed from ambient temperature to 700 °C. The crystal phases of samples were detected through XRD (Pananalytical X’Pert Pro, MPD, Almelo, The Netherlands) with high-intensity Cu Kα radiation. The sample was scanned from 5 to 50°. The results for the elements on the surface of LDH/P(AA-AMPS) gels and LDH were collected using XPS (Escalab 250Xi, Waltham, MA, USA). TEM observation was conducted using a JEM-ARM200CF (JEOL) instrument at 60 kV.

### 4.4. Self-Healing Performance Test

1. Self-healing time test

**Figure 10:**
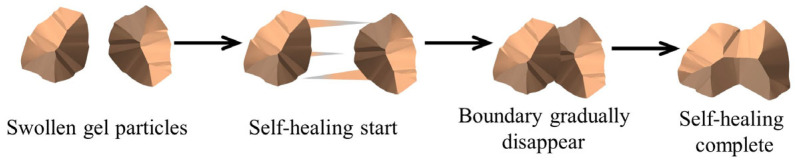
Adapted illustration of self-healing process.

The self-healing time of LDH/P(AA-AMPS) NC gels was explored by a bottle test [55]. The dried LDH/P(AA-AMPS) NC gel particles (10% concentration) were added to 5% bentonite mud, and stirred evenly, and then placed in the oven at different temperatures. The self-healing process of gels is shown in Figure 10. The morphology of gel was checked every 0.5 h until the boundary between the gels disappeared. After self-healing, the rheological properties, tensile properties, and adhesion properties of the self-healed gel were tested.

2. Rheological performance test

The rheology measurements were carried out using a rotary rheometer (MCR 320e, Anton Paar, Glaz, Austria) equipped with plate–plate geometry (diameter 25 mm, gap 2 mm). The strain amplitude scanning was performed from 0.1% to 1000%, under a constant frequency of 1 Hz. The frequency scan was tested in the range of 0.1 to 100 Hz and the strain was fixed at 1%.

3. Mechanical performance test

The tensile test of the self-healing gel was carried out by an electronic universal material testing machine (WH-50, Weiheng, Ningbo, China). For the uniaxial tensile test sample, the sample was cut into a rectangular shape with a length of 50 mm, a width of 15 mm, a thickness of 5 mm, a tensile rate of 60 mm/min, and a distance between the two fixtures of 25 mm.

4. Adhesion performance test

The self-healed gel was placed between two artificial cores with a diameter of 30 mm and maintained for 5 min by applying a load of 100 g to form good contact. Then, the core was clamped on the universal testing machine (WH-50, Weiheng, Ningbo, China) and loaded at a speed of 50 mm/min until the core was separated. Adhesion strength was calculated by the ratio of the maximum adhesion force to the adhesion area.

### 4.5. Swelling Behaviour Test

The LDH/P(AA-AMPS) NC gel particles with initial mass *M*_0_ were placed in an excessive plugging base mud (5% bentonite mud) and fully swelled at different temperatures. The mass *M_t_* of the gel was recorded at intervals, and the swelling ratio (*SR*) of the sample was determined by Equation (1) [38].

(1) $$SR=\frac{M_{t}-M_{0}}{M_{0}}$$

where *M*_0_ and *M_t_* are the mass of the sample at the initial time and time *t*, respectively.

### 4.6. Plugging Performance Test

**Figure 11:**
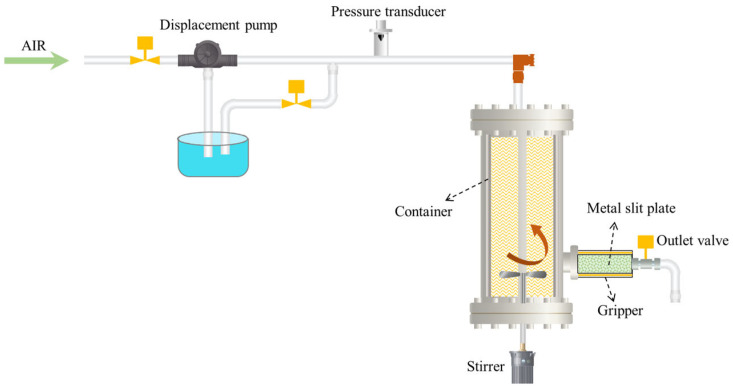
Schematic diagram of the plugging performance test experiment, adapted with permission from Ref. [14], 2022, Elsevier.

The plugging performance of LDH/P(AA-AMPS) NC gels was evaluated by a plugging evaluation device (Figure 11 and Figure S7) [14]. The working principle of the device is as follows: the dynamic process of well-bore circulation is simulated by placing the plugging slurry in the container that can be heated and stirred, and then the pressure is injected by the displacement pump. The LCM enters the metal slit plate in the gripper under the pressure difference force, and the pressure of the process is recorded in real time, so as to evaluate the pressure-bearing performance of LCM. Specifically, the gel particles were mixed with 5% bentonite mud to prepare a plugging mud with a particle concentration of 10%. The advection pump injected the plugging mud into the metal slit plate (15 cm in length, 3 mm at the inlet, and 1 mm at the outlet). Then, the metal slit plate was put into an aging tank containing 20 mL of bentonite mud, and kept at 90 °C for 3.5 h. Then, the metal slit plate was put into the gripper, the liquid outlet valve was opened, 2 L bentonite mud was injected into the container. The displacement pressure was set to 0.5 MPa, and it increased by 0.5 MPa every 2 min until the liquid continued to flow out of the liquid outlet. The maximum pressure corresponded to the pressure bearing capacity of the LDH/P(AA-AMPS) NC gels for the fracture.

## Figures and Tables

**Table 1:** Components for the preparation of different samples.

Samples	AA	AMPS	LDH	CaCl_2_
AC_0_L_0_	30	3	0	0
AC_0_L_3_	30	3	3	0
AC_6_L_0_	30	3	0	6
AC_6_L_3_	30	3	3	6
AC_3_L_3_	30	3	3	3
AC_9_L_3_	30	3	3	9
AC_6_L_1.5_	30	3	1.5	6
AC_6_L_4.5_	30	3	4.5	6

## Data Availability

The data presented in this study are available on request from the first author. The data are not publicly available due to ethical.

## Supplementary Materials

The following supporting information can be downloaded at: https://www.mdpi.com/article/10.3390/gels10020093/s1, Figure S1: High-resolution XPS spectra for AC_6_L_3_: (a) C 1s, (b) O 1s and Ca 2p; Figure S2: G′ and G″ of self-healed gels with different LDH additions; Figure S3: G′ and G″ of self-healed gels with different CaCl_2_ additions; Figure S4: G′ and G″ of AC_6_L_3_ self-healed gels at different temperatures; Figure S5: G′ and G″ of AC_6_L_3_ self-healed gels at brines; Figure S6: G′ and G″ of self-healed gels with different AC_6_L_3_ particle concentrations. Figure S7. The physical diagram of the plugging experimental device.
