# Supplementary material for: A Self-Healing Gel with an Organic–Inorganic Network Structure for Mitigating Circulation Loss
Source: Gels. 2024 Jan 25;10(2):93. doi: 10.3390/gels10020093 (PMC10887993; doi:10.3390/gels10020093)
Supplement: Supplementary file 1 [file gels-10-00093-s001.zip › gels-2843928-supplementary.pdf]

gels

MDPI

Article

A Self-Healing Gel with an Organic–Inorganic Network Structure for Mitigating Circulation Loss

Cheng Wang [1,2], Jinsheng Sun [1,2,*], Yifu Long [2,*], Hongjun Huang [2], Juye Song [3], Ren Wang [2], Yuanzhi Qu [2] and Zexing Yang [2]

[1] College of Chemistry and Chemical Engineering, Southwest Petroleum University, Chengdu 610500, China; wangchengscc@163.com
[2] CNPC Engineering Technology R&D Company Limited, Beijing 102206, China; huanghj_dri@cnpc.com.cn (H.H.); wangrdr@cnpc.com.cn (R.W.); quyz_dri@cnpc.com.cn (Y.Q.); yangzxdri@cnpc.com.cn (Z.Y.)
[3] CNPC Great Wall Drilling Engineering Company Limited, Beijing 102206, China; songjy.gwdc@cnpc.com.cn
* Correspondence: sunjsdri@cnpc.com.cn (J.S.); longyfdr@cnpc.com.cn (Y.L.)

Citation: Wang, C.; Sun, J.; Long, Y.; Huang, H.; Song, J.; Wang, R.; Qu, Y.; Yang, Z. A Self-Healing Gel with an Organic–Inorganic Network Structure for Mitigating Circulation Loss. *Gels* **2024**, *10*, 93. https://doi.org/10.3390/gels10020093

Academic Editor: Jordi Puiggali

Received11 January 2024
Revised: 22 January 2024
Accepted: 23 January 2024
Published: 25 January 2024

Copyright: © 2024 by the authors. Licensee MDPI, Basel, Switzerland. This article is an open access article distributed under the terms and conditions of the Creative Commons Attribution (CC BY) license (https://creativecommons.org/licenses/by/4.0/).

Figs. S1 through S6

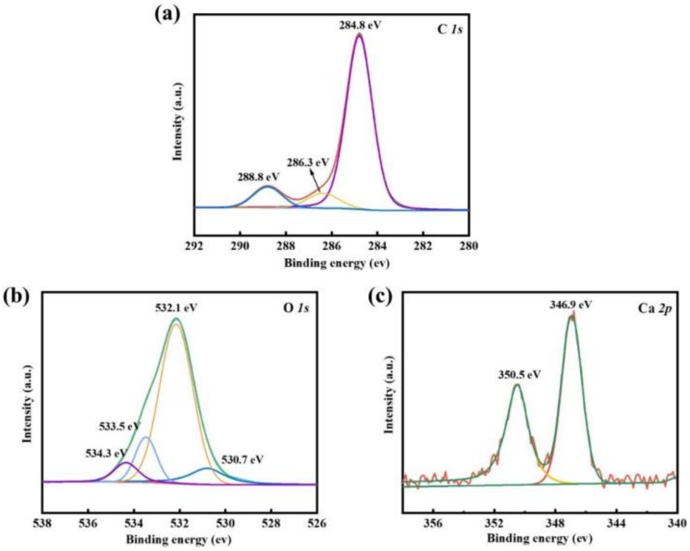

Figure S1. High-resolution XPS spectra for AC_6L_3: (a) C 1s, (b) O 1s and Ca 2p.

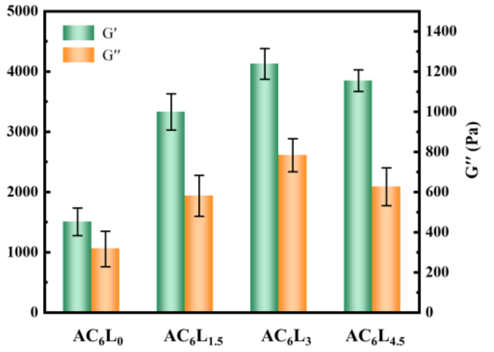

Figure S2. G' and G'' of self-healed gels with different LDH additions.

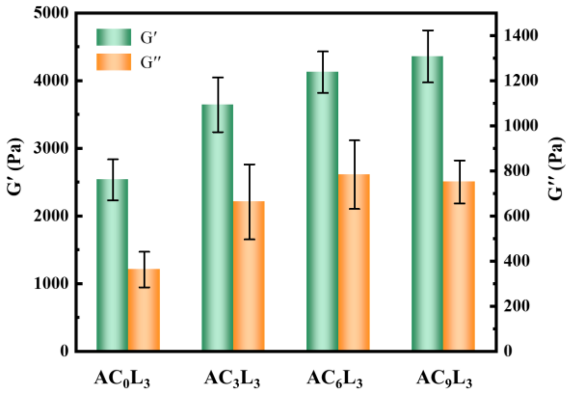

Figure S3. G' and G'' of self-healed gels with different $CaCl_2$ additions.

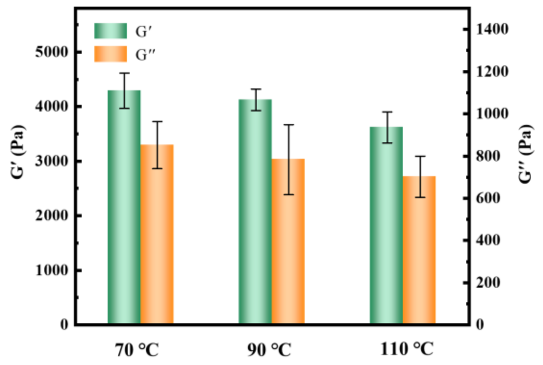

Figure S4. G' and G'' of AC_6L_3 self-healed gels at different temperatures.

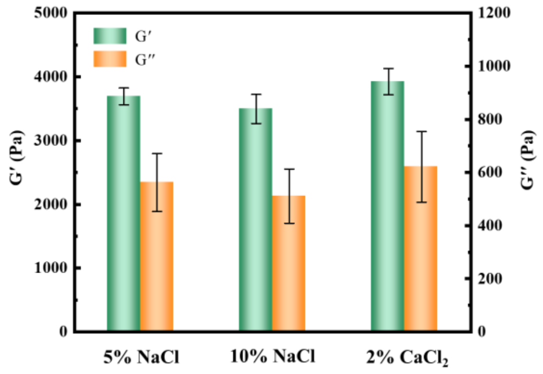

Figure S5. G' and G'' of AC_6L_3 self-healed gels at brines.

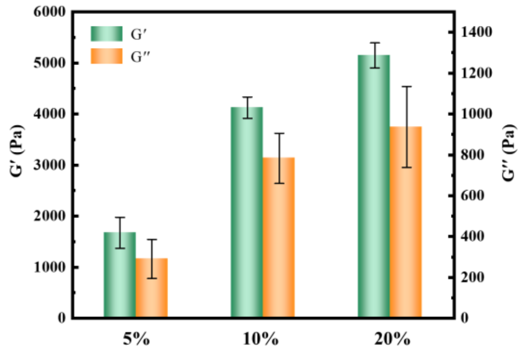

Figure S6. G' and G'' of self-healed gels with different AC_6L_3 particle concentrations.

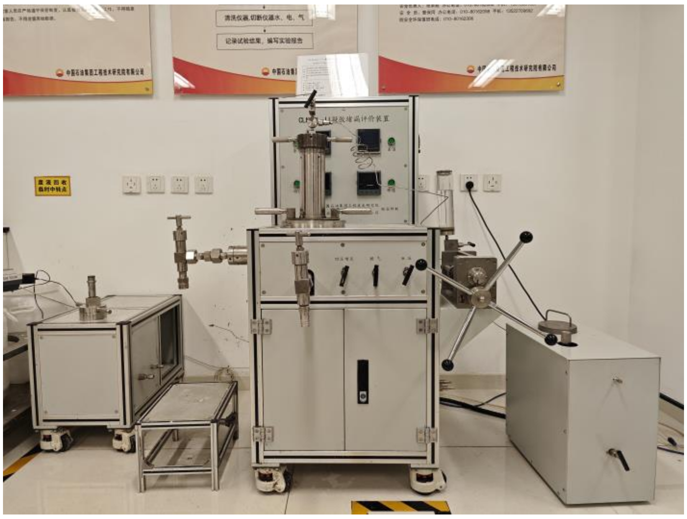

Figure S7. The physical diagram of the plugging experimental device.
